# The use of phage FCL-2 as an alternative to chemotherapy against columnaris disease in aquaculture

**DOI:** 10.3389/fmicb.2015.00829

**Published:** 2015-08-19

**Authors:** Elina Laanto, Jaana K. H. Bamford, Janne J. Ravantti, Lotta-Riina Sundberg

**Affiliations:** ^1^Centre of Excellence in Biological Interactions, Department of Biological and Environmental Science, University of JyvaskylaJyvaskyla, Finland; ^2^Department of Biosciences and Institute of Biotechnology, University of HelsinkiHelsinki, Finland

**Keywords:** aquaculture, disease, fish, *Flavobacterium columnare*, phage therapy

## Abstract

*Flavobacterium columnare*, the causative agent of columnaris disease in fish, causes millions of dollars of losses in the US channel catfish industry alone, not to mention aquaculture industry worldwide. Novel methods are needed for the control and treatment of bacterial diseases in aquaculture to replace traditionally used chemotherapies. A potential solution could be the use of phages, i.e., bacterial viruses, host-specific and self-enriching particles that can be can easily distributed via water flow. We examined the efficacy of phages to combat columnaris disease. A previously isolated phage, FCL-2, infecting *F. columnare*, was characterized by sequencing. The 47 142 bp genome of the phage had G + C content of 30.2%, and the closest similarities regarding the structural proteins were found in *Cellulophaga* phage phiSM. Under controlled experimental conditions, two host fish species, rainbow trout (*Oncorhynchus mykiss*) and zebrafish (*Danio rerio*), were used to study the success of phage therapy to prevent *F. columnare* infections. The survival of both fish species was significantly higher in the presence of the phage. Hundred percent of the zebrafish and 50% of the rainbow trout survived in the phage treatment (survival without phage 0 and 8.3%, respectively). Most importantly, the rainbow trout population was rescued from infection by a single addition of the phage into the water in a flow-through fish tank system. Thus, *F. columnare* could be used as a model system to test the benefits and risks of phage therapy on a larger scale.

## Introduction

In 2014, the WHO (2014) reported high rates of antimicrobial resistance in common disease-causing bacteria in all regions of the world. Concerns of antibiotic resistance have also brought attention to the large amounts of antibiotics used in animal production, also in aquaculture (Buschmann et al., 2012; Hollis and Ahmed, 2013). Antibiotics used in aquaculture may dissolve from the fish feed to the surrounding water (Cabello et al., 2013), thus enabling the development of antibiotic resistance in environmental microbes (Buschmann et al., 2012; Di Cesare et al., 2013). Indeed, concentrations of typically used antimicrobials (e.g., tetracycline) in marine sediments can be higher than the minimal inhibitory concentrations for most bacteria (reviewed in Cabello et al., 2013).

As aquaculture is the fastest growing industry for animal protein production in the world (Bostock et al., 2010), novel methods are needed for management of bacterial diseases. One option is phage therapy, which is already considered a feasible substitute for antibiotics in healthcare, livestock, and crop production (Smith and Huggins, 1983; Berchieri et al., 1991; Barrow et al., 1998; Nakai and Park, 2002; Jamalludeen et al., 2009; Santos et al., 2010; Abedon et al., 2011; Jones et al., 2012). Although the risk of development of phage resistance in the target bacteria needs to be considered, by e.g., using multiphage cocktails, phage therapy has great potential in the treatment of aquatic organisms. In contrast to antibiotics, phages are host-specific and self-enriching particles that can be can easily distributed via water flow within rearing units.

Columnaris disease (caused by *Flavobacterium columnare*, Bacteroidetes) is a significant problem in freshwater fish farming worldwide (Pulkkinen et al., 2010; Declercq et al., 2013). Currently, fish with columnaris disease are treated with antibiotics. Columnaris disease is a good candidate target for phage therapy since the disease is mainly external (on the fish gills, fins, and skin; Bernardet, 1997; Tripathi et al., 2005) and the bacteria transmit through water (Welker et al., 2005; Kunttu et al., 2009). The present study characterizes a phage, FCL-2 (Laanto et al., 2011), infecting *F. columnare* and examines how the phage treatment affects columnaris disease in rainbow trout (*Oncorhynchus mykiss*) and zebrafish (*Danio rerio*) under experimental conditions. We show that the phage can significantly alter the outcome of infection by *F. columnare*. In addition to proving its functionality in a specific system, this study highlights the use of *F. columnare* and its phage as a model for the implementation of phages as a treatment, namely phage therapy, in a real disease context in a real host. This system could also be used to estimate the possible (environmental) side effects of the method in practice, such as the effect of the presence of fish host and antibiotic treatments on evolution of phage resistance, or the effect of phage additions on natural bacterial community.

## Materials and Methods

### Bacteria and Phage

*Flavobacterium columnare* strain B185 and phage FCL-2 were both isolated from a fish farm rearing mainly salmonid fingerlings during a columnaris outbreak in 2008 (Laanto et al., 2011). Bacteria and phage were cultured in Shieh medium (Decostere et al., 1997), without tobramycin (24°C, 110 rpm) and stored in 10% glycerol and 10% fetal calf serum at –80°C. The optical density of B185 was measured at 570 nm to determine the colony forming units (CFU) per mL based on our unpublished analyses. For plaque assays the “double layer agar” –method (Adams, 1959) was used as follows: melted top agar (0.7%) including 300 μL of host bacterium and 100 μL of phage dilution was poured on Shieh agar and grown for 48 h at room temperature. Phage stocks were prepared from Shieh agar plates with confluent lysis by adding 5 mL of Shieh-medium on top of a plate and incubated at 6°C for 6 h. The lysate was collected, filtered, and stored at +4°C and for longer periods at –80°C with 10% glycerol. Phage morphology was studied previously (Laanto et al., 2011), but a higher quality transmission electron microscope was obtained for this study (Jeol JEM-1400 at 80 kV). For transmission electron microscopy (TEM) analysis, the phage was pelleted (Beckman coulter L-90K, 70 Ti-rotor, 25 000 × *g*, 2 h, +4°C) and washed twice with 0.1 M ammonium acetate, pelleted as above and suspended in 0.02 M potassium phosphate. The phage was spotted on a copper-coated grid for 2 min and excess suspension was dried with filter paper. Ten microliters of 1% phosphotungstate, at pH 6.5, were applied on the grid for 1 min and the grid was dried with filter paper.

### Phage Adsorption and Stability

The phage adsorption rate was measured in three replicates in 1 mL of Shieh medium with logarithmic phase B185 cells (1.4 × 10^8^ CFU), with a doubling time of approximately 10 h at RT (Zhang et al., 2014). Phage (2.2 × 10^4^ PFU, resulting to a MOI of 1.6 × 10^-4^) was added to each tube and tubes were shaken and then left to stand at RT; the control contained only the medium without cells. The bacterium-phage mixture was pelleted after 1, 5, 10, 20, 40, and 60 min by centrifugation (15 000 × *g*) and the PFU mL^-1^ of the free phage particles in the supernatant at each time point was determined to calculate the corresponding number of adsorbed phage particles. The adsorption rate was determined as the exponential decrease of free phage particles during the time of incubation.

The phage ability to remain infective in different buffers was tested by diluting FCL-2 lysate (original 1.2 × 10^11^ PFU mL^-1^) at a ratio of 1:100 in Shieh medium, into 20 mM Tris-HCl (pH 6.1, 7.6, and 8.1) and 20 mM potassium phosphate (pH 6.0, 7.2, and 8.0). All dilutions were kept at 6°C (and one dilution in Shieh medium at room temperature) for 6 weeks and PFU mL^-1^ was determined after 1 and 6 weeks as described above.

### Phage Genome Sequencing

The protocol developed by Santos (Santos, 1991), with slight modifications, was used to isolate phage DNA from the phage lysate. Briefly, phage particles were precipitated by adding 40 mM ZnCl_2_ and incubating for 5 min, followed by pelleting of the phage precipitate (15 000 × g, 5 min). DNA was purified using a GeneJET^TM^ Genomic DNA isolation kit column (Fermentas). The phage genome was sequenced in two platforms (Ion Torrent PGM with 100 bp kit and commercially with Roche 454 at LGC Genomics, Germany) and the data were combined because neither of the methods resulted in whole genome sequence. Initially, in-house Ion Torrent run was used for *de novo* assembly. Since the assembly did not yield a single contig, we decided to try to improve the assembly by using also commercial paired-end 454 sequencing. All analyses were done using GS *De Novo* Assembler version 2.9 (454 Life Sciences; Roche) which uses Overlap Layout Consensus (OLC) methodology. Open reading frames (ORFs) were predicted using Glimmer and GeneMarkS, and similar sequences were searched from databases using BLAST (Altschul et al., 1990) with Geneious version 7.1 (created by Biomatters Ltd).

### Fish Experiments with Zebrafish (*Danio rerio*) and Rainbow Trout (*Oncorhynchus mykiss*)

Fish experiments were conducted according to the Finnish Act on Use of Animals for Experimental Purposes, under permission EASVI-2010-05569/Ym-23 granted for L-RS by the National Animal Experiment Board at the Regional State Administrative Agency for Southern Finland. Unsexed, adult, disease-free zebrafish (*D. rerio*) were obtained from the core facilities and research services of Tampere (Tampere University, Finland). Prior to the experiments, the zebrafish were maintained in 250-L aquaria containing aerated ground water at 25°C. Rainbow trout (*O. mykiss*) fry with no previous contact with *F. columnare* were obtained from a fish farm in central Finland (approximate weight in the experiments 0.57 g). Prior to the experiments, the rainbow trout were maintained in aerated ground water at 17°C in 250-L flow-through aquaria. For the infection experiments, the water temperature of rainbow trout was gradually elevated to 24°C over 7 days. Two infection methods were used. Zebrafish were infected by applying *F. columnare* directly to the experimental aquaria, resulting in continuous infection. The bacterial levels used for infection were chosen based on our previous studies on rainbow trout (see, e.g., Kunttu et al., 2009) and on zebra fish (Laanto et al., 2012; Zhang et al., 2014). Rainbow trout populations were immersed in water containing the bacterium for 2 h, after which fish were transferred to experimental aquaria. The fish were monitored in 2-hour intervals in the zebrafish experiment and in 12-hour intervals in the rainbow trout population experiment. Fish aquaria had running numbers and were mixed after addition of phage. Morbid fish that did not respond to stimuli were considered dead and removed from the experiment. In all experiments, fin cultivations on Shieh agar supplemented with tobramycin were taken from moribund fish to determine the presence/absence of *F. columnare* on the fish.

### Measuring the Effect of Phage Addition to a Flow-Through System with Rainbow Trout Population Infected with *F. columnare*

For infection, the rainbow trout fry (*n* = 260) were divided into 13 groups of 20 fish, and placed in 3-L aquaria containing 2 L of ground water. Nine fish grpups were exposed to 3 × 10^6^ CFU mL^-1^ of *F. columnare* (calculated from optical density at 570 nm based on the data from unpublished experiments) and four control groups to sterile Shieh medium for 2 h under aeration at 24.2°C. After the challenge, fish populations were transferred to experimental aquaria containing 2 L of ground water with a constant inflow of fresh water (∼0.6 L min^-1^, resulting in an approximately 3.3 min turnover time of the whole water body) and aeration, at a temperature ranging from 23.7 to 24.4°C during the experiment. The phage was added to experimental aquaria after fish transfer with phage-to-bacterium ratios of 1:1 (three aquaria) and 10:1 (three aquaria) compared to infection dose, and the highest amount was added to two control aquaria with fish exposed to Shieh medium. During phage addition, the water flow was stopped for 60 min for all of the aquaria. Fish were monitored for 7 days in 12-hour intervals.

During the experiment, water samples were taken after 24 and 48 h from the start of the experiment to determine the number of *F. columnare* cells (CFU mL^-1^) and FCL-2 phages (PFU mL^-1^) in the water. Samples were taken from two aquaria from the following treatments: phage-to-bacterium ratios 1:1 and 10:1, phage control, and bacterial control. Water samples were diluted and plated on a Shieh agar plate supplemented with tobramycin (Decostere et al., 1997) and *F. columnare* colonies were counted after a 48-hour incubation at room temperature. PFU were determined from the same dilution series by culturing 300 μL of host bacterium and phage sample dilutions in Shieh soft agar, as described above.

### Effect of Phage Addition in Experimental *F. columnare* Infection of Individual Zebrafish

Zebrafish (*n* = 60) were placed individually in 1-L aquaria containing 500 mL of ground water (24.4°C). Fish (*n* = 40) were infected in continuous exposure with 5 × 10^4^ CFU mL^-1^ of *F. columnare*. The phage was added to 20 of these aquaria directly after infection at a phage-to-bacteria cell ratio of 1:1. Phage-to-bacteria ratio from 0,1:1 to 100:1 was tested in preliminary studies. There was no clear difference between ratios of 1:1 and 1:100, therefore the lowest ratio that had an effect to fish survival was chosen. Control fish received sterile Shieh medium (*n* = 10) and phage only (*n* = 10). The total volume addition to each aquarium was 500 μL.

### Statistical Analysis

Fish survival in experimental infections was analyzed using IBM-SPSS statistics 20. The fish survival was analyzed with Kaplan–Meier survival analysis.

### Phage Genome Accession Number

The nucleotide sequence of the FCL-2 phage genome has been deposited to GenBank (accession number KM873719).

## Results

### Phage Characterization and Genome Analysis

**FIGURE 1:**
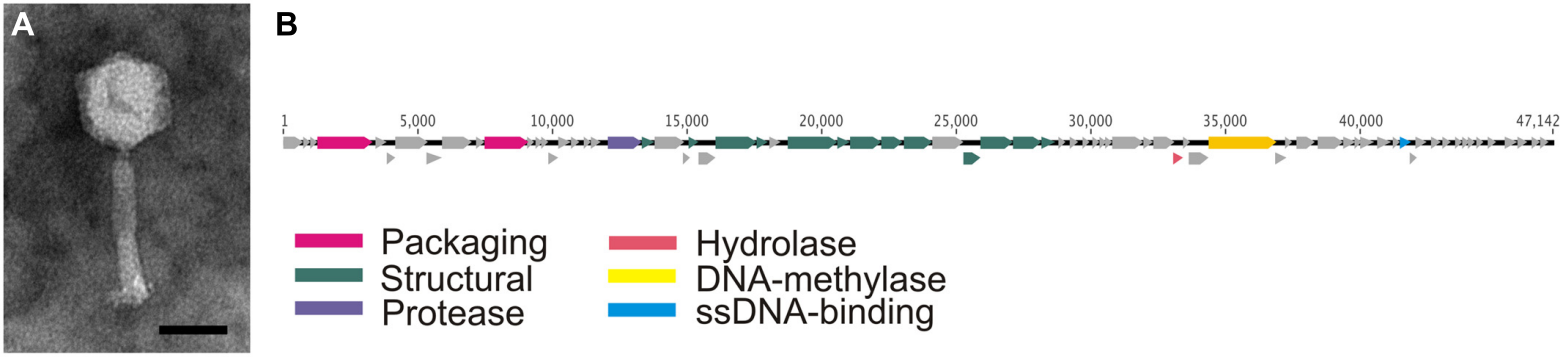
**(A)** Phage FCL-2 viewed under transmission electron microscopy (TEM; scale bar 40 nm). **(B)** Graphic representation of the genome organization of FCL-2 with colors indicating the putative functions for open reading frames (ORFs) as shown in the figure.

Phage FCL-2 has been previously determined as a member of *Myoviridae* (Laanto et al., 2011). TEM analysis revealed the diameter of the icosahedral capsid to be 55–60 nm. The tail was approximately 85 nm long. A prominent neck was visible and tail fibers could be seen (**Figure 1**). Enrichment of the phage was done on plates because despite of optimization of culture conditions and parameters, the cells infected with phage have not lysed under liquid culture.

Sequence of the FCL-2 genome was obtained by combining Ion Torrent and 454 data. Using both data sets (total number of sequences: 34680; total number of bases: 6155764) we were able to *de novo* assemble a single 47 142 bp long contig with coverage of 125.9 and G + C content of 30.2%. Seventy-four ORFs all in forward direction were predicted with Glimmer and GeneMarkS. For 18 of these, a function was predicted by BLAST search and 32 were assigned as hypothetical proteins, leaving 24 ORFs (32.4%) without any significant homologs in BLAST search (as of February 11, 2015). ORFs with predicted functions included proteins involved in packaging (terminase and phage portal protein; **Figure 1** and Supplementary Table S1) and a protease, a DNA-methylase, and an ssDNA-binding protein. No direct lysis genes were detected although a match to a hydrolase gene was received: This could possibly have a role in the lysis of the cell. Most of the predictions corresponded to structural proteins (e.g., tail and tail sheath protein) located from 13 338 to 28 169 bp in the genome. These structural proteins were found to match the *Cellulophaga* phage phiSM and matches to seven other *Cellulophaga* phages were also obtained from these genes. Further, from the predicted hypothetical proteins, one matched the *Flavobacterium* phage 11 b and two hits were linked to the genome of prophage 6H (Castillo et al., 2013). These two prophage 6H-related proteins, IbrA and IbrB, are also found in *F. psychrophilum* (the host of 6H) and in *F. columnare* genomes. Additionally, two ORFs upstream from the IbrAB complex were identical to genes found in *F. columnare* ATTC 49512 genome (accession number in NCBI: NC_016510.2, FCOL_05310 (WP_014165166.1), and FCOL_05310 (WP_014165168.1).

### Phage Adsorption Test and Stability

In the adsorption test, 27% of phage FCL-2 had adsorbed on the cells of *F. columnare* B185 after 1 min and 41% after 10 min. After 1 h, 50% of the added phages had adsorbed, resulting into an estimated rate of adsorption being 0.5 h^-1^. The stability of FCL-2 titers was not affected by storage temperature (**Table 1**). After a 6-week period, the titer of the lysate stored at room temperature was less than that of the lysate stored at 6°C. The overall titer over 6 weeks decreased 10-fold when stored in Shieh medium or Tris-HCl buffer, and 100-fold when stored in the presence of potassium phosphate.

**Table 1:** Phage FCL-2 stability as plaque forming units (PFU) per mL during 6-week storage.

	RT	6°C						
			Tris-HCl			Potassiumphosphate		
Week	Shieh	Shieh	pH 6	pH 7,5	pH 8	pH 6,0	pH 7,2	pH 8,0
**1**	9.7 × 10^10^	1.5 × 10^11^	1.8 × 10^11^	2 × 10^11^	3.3 × 10^11^	6.3 × 10^10^	8.5 × 10^10^	9.7 × 10^10^
**6**	3.6 × 10^9^	1 × 10^10^	1.5 × 10^10^	1.6 × 10^10^	1.3 × 10^9^	1.9 × 10^9^	4.3 × 10^9^	5 × 10^9^

*The original phage titer at the beginning of the experiment was 1.2 × 10^11^ PFU mL^-1^. All experiments were performed at 6°C, except for Shieh treatment, which was performed at room temperature (indicated as RT).*

### Effect of Phage Addition on Survival of the Rainbow Trout Population in a Flow-Through System

**FIGURE 2:**
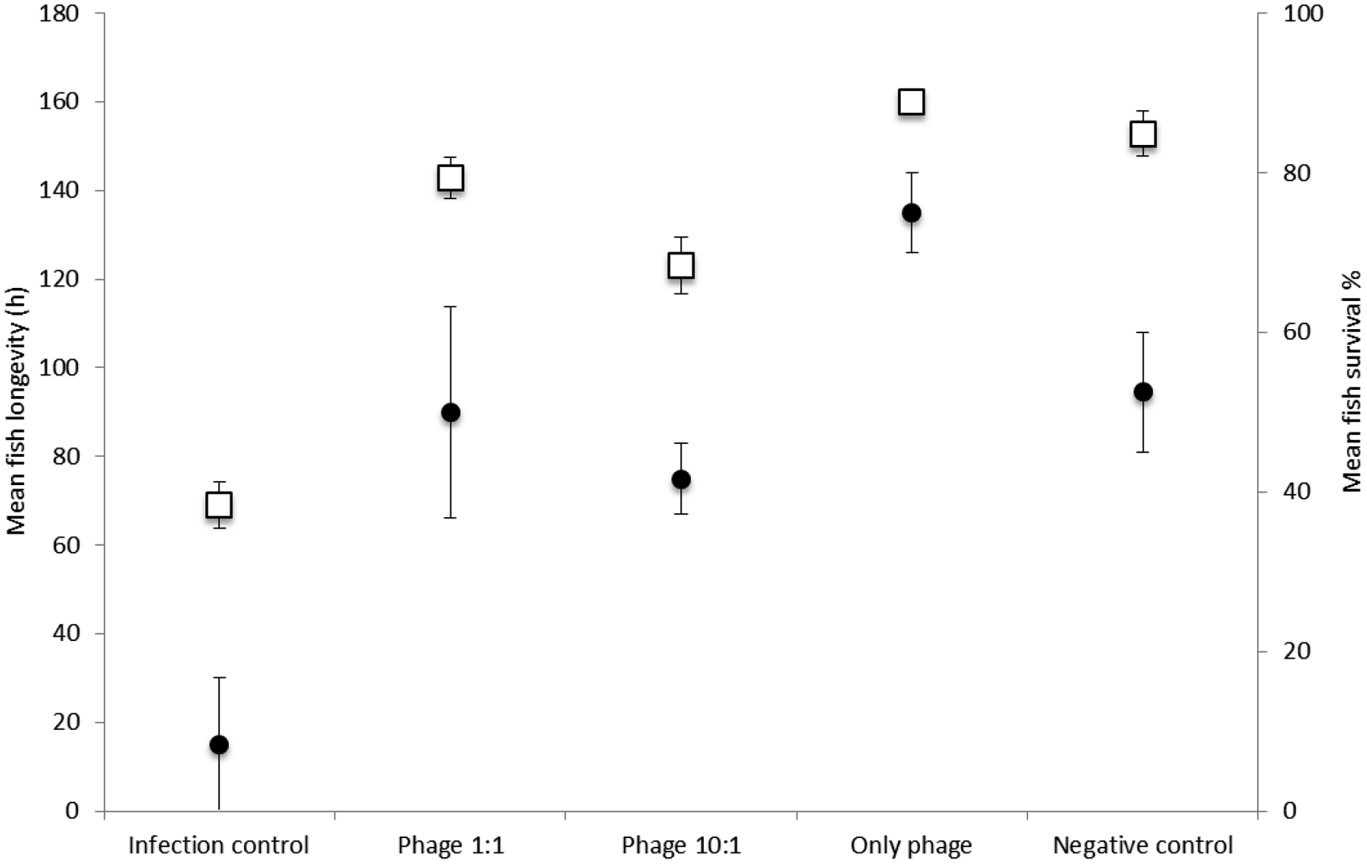
**Fish longevity in hours (squares, left axis; mean ± SE) and survival percentage (dots, right axis; mean ± SE) of rainbow trout after exposure to *Flavobacterium columnare* B185 and phage FCL-2 in three replicate fish populations (*n* = 20).** Fish were infected by immersion (2 h) and phage was added into experimental aquaria after bacterial challenge (in phage-to-bacterium ratios of 1:1 and 1:10). See **Table 2** for statistical comparisons.

To mimic real life conditions at a fish farm, the phage efficiency was tested in pre-infected populations of rainbow trout fingerlings under continuous water flow. The fish were given phage immediately after bacterial exposure (2-hour immersion in bacteria). A significant increase in survival of fish following experimental phage treatments was observed (**Figure 2**, **Table 2**). At the end of the experiment (7 days post infection), the mean survival of the fish infected only with *F. columnare* (infection control) was 8.3%. Phage treatment protected the fish from infection and resulted in a 41.7% (ratio 10:1) and 50% (ratio 1:1) mean survival (*P* < 0.001 for all pairwise comparisons against phage-treated groups and infection control; **Table 2**). Fish in the negative control group (not infected) survived 80–100 h post infection, after which background mortality was observed due to the experimental conditions, rather than the treatments. Nevertheless, in pairwise comparisons, survival of fish in the negative control group (no bacteria, no phage) did not differ from those receiving phage treatments after bacterial infection.

**Table 2:** Statistics of pairwise comparisons in fish survival in phage therapy experiments against *Flavobacterium columnare* in rainbow trout (*Oncorhynchus mykiss*) and zebrafish (*Danio rerio*) using Kaplan–Meier survival analysis, pairwise log rank Mantel Cox test.

Experiment and overall statistics	Treatment	Statistics for pairwise comparisons		
		Infection control	Phage 1:1	Phage 1:10
Rainbow trout, population	Negative control	χ^2^ = 49.272, *p* < 0.001	NS	NS
	Phage 1:1	χ^2^ = 57.235, *p* < 0.001		
	Phage 1:10	χ^2^ = 31.824, *p* < 0.001	NS	
	Only phage	χ^2^ = 64.390, *p* < 0.001	χ^2^ = 6.741, *p* = 0.009	χ^2^ = 12.777, *p* < 0.001
Zebra fish, continuous infection	Negative control	χ^2^ = 26.199, *p* < 0.001	χ^2^ = 4.9542, *p* < 0.026	
	Phage 1:1	χ^2^ = 17.853, *p* < 0.001		

*P-values greater than 0.05 are designated as non-significant (NS).*

In the rainbow trout trial, all diseased fish treated with bacteria only were positive for *F. columnare* rhizoid morphology according to fin samples. The cultivations from the phage-treated fish showed mainly a rough colony morphology (possibly due to the presence of phage in the sample, see Laanto et al., 2012) with occasional rhizoid colonies. All fish not exposed to bacteria were free of *F. columnare*.

### Effect of Phage Addition to the Survival of Zebrafish in a Continuous Infection Model

**FIGURE 3:**
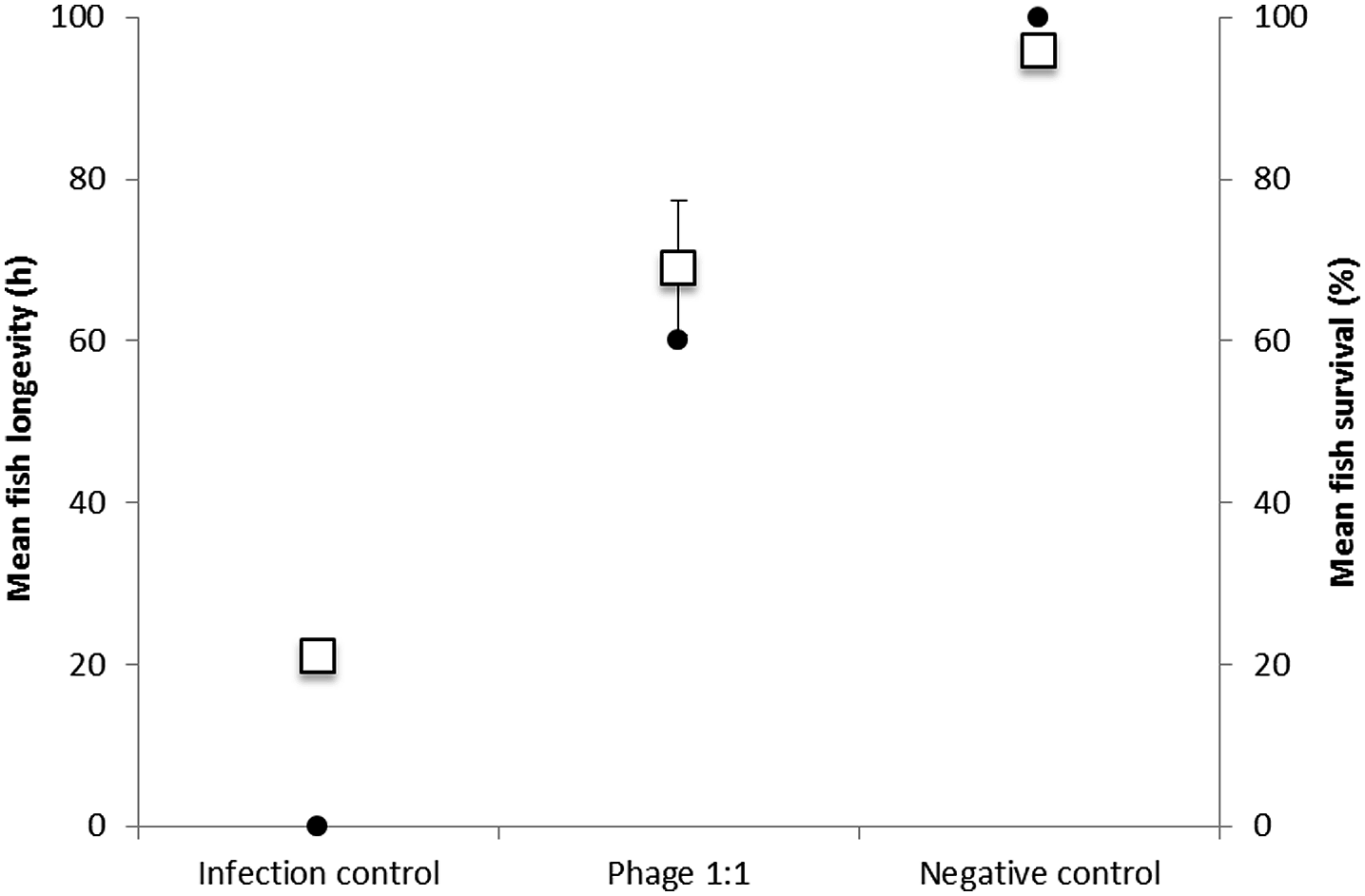
**Fish longevity in hours (squares, left axis; mean ± SE) and survival percentage (dots, right axis; mean ± SE) of the zebrafish after exposure to *F. columnare* strain B185 (infection control), B185 and phage FCL-2 (1:1), and to growth medium or phage only (negative control).** See **Table 2** for statistical comparisons.

To test if zebrafish can potentially be used as a model system to study phage–bacterium interactions, including the host, instead of rainbow trout (due to the poor availability of disease-free fingerlings and sensitivity to laboratory conditions), an infection experiment was performed with individual continuous exposure to bacterium and phage. The survival of the non-infected controls in the experiment was 100% versus 0% in the infection control group treated only with bacteria. Phage addition significantly increased fish survival, resulting in 60% of survival (**Figure 3**, **Table 2**).

In bacterial culture samples taken from zebrafish, all fish treated with bacteria only were positive for the virulent rhizoid colony morphology of *F. columnare*. Three of the six dead fish that were treated with phage were positive for *F. columnare* non-virulent rough type (possibly due to the presence of phage in the sample, see Laanto et al., 2012). Further, all the fish in bacteria-free controls were negative for *F. columnare*.

### Phage and Bacterial Counts during Experiments

Water samples were taken from the experimental aquaria during the rainbow trout experiment. During the rainbow trout population test in a flow-through system, the FCL-2 phage was isolated from the water in at least 1 × 10^4^ PFU mL^-1^ even after 24 and 48 h post infection. This indicates that the phage was able to replicate in the system and was not washed away by the constant water flow. The maximum *F. columnare* counts in the population experiment were 8.8 × 10^2^ CFU mL^-1^ in the bacterial infection control and from the phage-treated aquaria, 2.7 × 10^2^ CFU mL^-1^. All of the colonies counted were of the rhizoid morphotype. No *F. columnare* was isolated from the water samples of the bacteria-free control group.

## Discussion

Herein, the first attempts to apply phage therapy against columnaris disease, caused by *F. columnare*, are presented. The first genome of a phage infecting *F. columnare* (FCL-2) was sequenced and characterized for its ability to increase fish survival in the presence of the pathogen. Structural proteins of FCL-2 were related to those of *Cellulophaga* phages. *Cellulophaga*, a member of the Bacteroidetes group, is a close relative to Flavobacteria (Holmfeldt et al., 2007). Using FCL-2, it was shown that phages have great potential in controlling columnaris disease in small-scale experimental systems, but also in rainbow trout populations in flow-through tanks.

Thorough characterization of a phage is essential for its therapeutic use in order to avoid unwanted interactions that might increase the virulence of its bacterial host (Gill and Hyman, 2010). Genome sequencing revealed that a third of the phage ORFs’ had no homologs in the database, which is typical for environmental phage genomes (Hurwitz and Sullivan, 2013). Only few ORFs of the FCL-2 phage were related to *Flavobacterium* phages, most probably due to the low number of these phage genomes available. A gene possibly related to host lysis (hydrolase) was identified in the FCL-2 genome. Clear signs of possible integration to host genome (integrases) or repressors were absent, and lysogeny of the host after exposure to the phage has not been detected (Laanto et al., 2012). A few host hypothetical proteins were identified, including proteins of unknown function, IbrA and B (Supplementary Table S1). In the genome analysis of *F. psychrophilum* prophage 6H it was hypothesized that these two proteins could be possible virulence factors due to their relation to the IbrAB complex in *Escherichia coli* (Castillo et al., 2013). This complex is possibly involved in host–pathogen interactions and related to resistance to serum and Ig-binding (Sandt and Hill, 2000). Interestingly, IbrA and IbrB and the two ORFs located upstream can also be found in the sequenced genome of *F. columnare* ATTC 49512, indicating a common origin for these regions. Clearly, more studies are needed to understand the phage life cycles and phage–bacterium interactions in *F. columnare*.

The results from phage therapy trials in animal production (e.g., in poultry, swine, and cattle) have given encouraging results (Smith and Huggins, 1983; Berchieri et al., 1991; Barrow et al., 1998; Jamalludeen et al., 2009; Santos et al., 2010), similar to phage therapy studies against fish diseases (Nakai and Park, 2002; Park and Nakai, 2003; Higuera et al., 2013; Khairnar et al., 2013). The interest on efficiency of phages against *F. psychrophilum* (Stenholm et al., 2008; Castillo et al., 2012) and *F. columnare* (Laanto et al., 2012; Prasad and Kumar, 2012) has been recently increasing, reflecting the global concern toward flavobacterial pathogens in aquaculture. Generally, the efficiency of a phage as a potential therapeutic is measured by its capacity to destroy bacterial cells at the site of bacterial infection (Levin and Bull, 2004). Previous studies have administered the phages via fish feed and injection (Nakai et al., 1999; Park and Nakai, 2003; Castillo et al., 2012; Kim et al., 2015), which could be laborious when handling large numbers of fish. However, as columnaris disease is mainly external, phages could be also applied directly in the water. Herein, a significant increase was observed in the survival of zebrafish and rainbow trout when the phage was applied in the water shortly after bacterial exposure. When the phage was added into aquaria directly after bacteria, zebra fish survival increased most likely due to a decrease in bacterial infection dose. In contrast, in the rainbow trout experiment, bacteria were allowed to colonize the fish for 2 h prior to placing them in the experimental flow-through tanks where the phage was added. Hence, the increase in fish survival was likely caused by the association of phage and bacteria on fish surfaces rather than via direct effects on infection dose, although the presence of phage in the water is also likely to reduce the transmission of disease between individual fish. Surprisingly, the phage was isolated from the tank water 2 days post infection, indicating that it can persist and replicate in the tanks despite water flow when host bacteria are present. Furthermore, the FCL-2 titer remained stable over a 6-week period, indicating promising persistence and storage qualities required for the development of therapeutic applications in aquaculture.

Bacteria have developed multiple mechanisms against phage infection (Samson et al., 2013); nevertheless, in the continuous arms race between a phage and its host, phages co-evolve to outcompete these mechanisms. Therefore, resistance to a certain phage may not be a problem in phage therapy (Örmälä and Jalasvuori, 2013), especially as maintaining resistance can be costly for bacteria (Laanto et al., 2012, reviewed in Koskella and Brockhurst, 2014). Indeed, our previous results suggest that following strong phage selection *F. columnare* changes colony morphotype (Laanto et al., 2012). Rapid development of phage resistance may thus not be a problem in *F. columnare* as the resistant cells are not capable of causing disease in fish (Laanto et al., 2012). However, the phage-resistant rough morphotype has not been isolated from disease outbreaks, indicating that environmental conditions outside the laboratory influence the evolution or persistence of phage resistance of *F. columnare* at fish farms. Therefore, more studies on phage resistance mechanisms in this fish pathogen are needed.

The phages isolated against *F. columnare* strains are very host specific when compared to *Flavobacterium* sp. phages from fish farms and outside the fish farming environment (Laanto et al., 2011). This is promising as it suggests that other bacterial species would not be affected. As Meaden and Koskella (2013) indicate, the possibility that other species of bacteria might be influenced should be taken into account. Although the present study shows that phages significantly increased rainbow trout survival in flow-through water, the condition most relevant for fish farming, there are still many aspects that need to be optimized before reaching production scale. First, the population structure of *F. columnare* at farms is complex and several strains co-occur and may co-infect fish (Suomalainen et al., 2006; Kunttu et al., 2012). Therefore, multiple phages and their associations with the host bacteria need to be characterized and tested for efficiency in mixed infections and to prevent evolution of phage resistance. Second, although phage administration through water is relatively simple, the water masses that need to be maintained saturated are large and phage will be quickly diluted. Since our data suggest that phages are able to persist in flow-through tanks, this challenge may not be insurmountable, especially if phages are used to reducing the infective bacterial dose in the tanks. Similarly, the timing of treatment needs to be optimized, as fish in the rearing units are often at different stages of infection during an outbreak. Problems related to enrichment of phage FCL-2 in liquid culture could be a severe problem concerning the larger scale production of phages for phage therapy, at least when using this specific phage. Also, *F. columnare* is a slow-growing bacterium, with a doubling time approximately of 10 h (Zhang et al., 2014), which could bring extra challenges for production scale. Further, the optimal dose of phage (phage–bacterium ratio) is crucial for treatment efficacy and needs to be evaluated, a fact not thoroughly considered in early phage therapy trials (Carlton, 1999). Despite these challenges in the application of phage therapy in aquaculture, it would be worthwhile to assess whether phage therapy could be combined with other treatments. If phage therapy could decrease the bacterial infection dose during disease outbreaks and reduce the risk of transmission, the need for antibiotic treatment could be delayed or targeted to smaller groups of infected individuals. In the long run, this would lead to a reduction in antibiotics use in aquaculture.

## Conclusion

The phage therapy has potential as an efficient and sustainable disease management method in the case of columnaris disease and aquaculture in general. It is shown herein that *F. columnare* and its phage form a good model for the study of phage therapy as the disease and treatment can be studied in the relevant hosts. This model could be used to study the ecological and evolutionary effects of phage therapy. Further, aquaculture conditions with high population densities of fish hosts can thus be replicated in the laboratory. Finally, using zebrafish as a model host could extend the benefits of the experimental system to include the fish host immune response during phage therapy.

## Author Contributions

EL, JB, and L-RS designed the study. EL performed the experiments. EL, JR, and L-RS analyzed the data. EL and L-RS prepared the tables and figures and wrote the manuscript. All authors edited the manuscript.

## Conflict of Interest Statement

The authors declare that the research was conducted in the absence of any commercial or financial relationships that could be construed as a potential conflict of interest.
