# Supplementary material for: The use of phage FCL-2 as an alternative to chemotherapy against columnaris disease in aquaculture
Source: Front Microbiol. 2015 Aug 19;6:829. doi: 10.3389/fmicb.2015.00829 (PMC4541368; doi:10.3389/fmicb.2015.00829)
Supplement: Supplementary file 1 [file Data_Sheet_1.PDF]

Supplemental Table 1. ORF identification of FCL-2 genes.

<table>
<tr><th>ORF</th><th>Start</th><th>End</th><th>Length</th><th>Putative function</th><th>Best hit</th><th>Number of hits (max 100)</th><th>E-value, Score, Bitscore, Identities</th></tr>
<tr><td>orf00001</td><td>18</td><td>725</td><td>708</td><td>Hypothetical protein</td><td>hypothetical protein [Muricauda sp. MAR_2010_75]</td><td>3</td><td>E-value = 4.04e-26, Score = 279, Bitscore = 112.079, Identities = 71/222 (31%)</td></tr>
<tr><td>orf00002</td><td>805</td><td>1,008</td><td>204</td><td>NO RESULTS</td><td></td><td></td><td></td></tr>
<tr><td>orf00003</td><td>1,015</td><td>1,275</td><td>261</td><td>NO RESULTS</td><td></td><td></td><td></td></tr>
<tr><td>orf00004</td><td>1,288</td><td>3,327</td><td>2,040</td><td>Terminase</td><td>phage terminase [Cellulophaga phage phiSM] >gi|451988688|gb|AGF91164.1| terminase [Cellulophaga phage phi3:1] >gi|451989197|gb|AGF91668.1| terminase large subunit [Cellulophaga phage phi47:1] >gi|460109484|gb|AGH07751.1| phage terminase [Cellulophaga phage phiSM] >gi|514341476|gb|AGO47735.1| phage terminase large subunit [Cellulophaga phage phi3ST:2] >gi|514341946|gb|AGO48199.1| phage terminase large subunit [Cellulophaga phage phiSM] >gi|514343005|gb|AGO49243.1| phage terminase large subunit [Cellulophaga phage phi38:2] >gi|514343086|gb|AGO49323.1| phage terminase large subunit [Cellulophaga phage phi3:1] >gi|514343509|gb|AGO49741.1| phage terminase large subunit [Cellulophaga phage phi47:1]</td><td>100</td><td>E-value = 0, Score = 1501, Bitscore = 582.793, Identities = 294/680 (43%</td></tr>
<tr><td>orf00005</td><td>3,424</td><td>3,828</td><td>405</td><td>NO RESULTS</td><td></td><td></td><td></td></tr>
<tr><td>orf00006</td><td>3,825</td><td>4,148</td><td>324</td><td>Hypothetical protein</td><td>hypothetical protein [Flavobacterium columnare] >gi|365960115|ref|YP_004941682.1| hypothetical protein FCOL_05320 [Flavobacterium columnare ATCC 49512] >gi|365736796|gb|AEW85889.1| hypothetical protein FCOL_05320 [Flavobacterium columnare ATCC 49512]</td><td>8</td><td>E-value = 3.61e-59, Score = 479, Bitscore = 189.119, Identities = 92/107 (85%)</td></tr>
<tr><td>orf00007</td><td>4,145</td><td>5,356</td><td>1,212</td><td>Hypothetical protein</td><td>hypothetical protein [Flavobacterium columnare] >gi|365960113|ref|YP_004941680.1| hypothetical protein FCOL_05310 [Flavobacterium columnare ATCC 49512] >gi|365736794|gb|AEW85887.1| hypothetical protein FCOL_05310 [Flavobacterium columnare ATCC 49512]</td><td>100</td><td>E-value = 6.89e-165, Score = 1238, Bitscore = 481.485, Identities = 241/391 (61%)</td></tr>
<tr><td>orf00008</td><td>5,340</td><td>5,894</td><td>555</td><td>Hypothetical protein (IbrB)</td><td>nuclease [Flavobacterium psychrophilum] >gi|150024891|ref|YP_001295717.1| Protein of unknown function IbrB [Flavobacterium psychrophilum JIP02/86] >gi|526120163|ref|YP_008320442.1| hypothetical protein [Flavobacterium phage 6H] >gi|149771432|emb|CAL42901.1| Protein of unknown function IbrB [Flavobacterium psychrophilum JIP02/86] >gi|511624410|gb|AGN89411.1| hypothetical protein [Flavobacterium phage 6H] >gi|697783246|gb|AIT65708.1| chromosome partitioning protein ParB [Flavobacterium psychrophilum]</td><td>100</td><td>E-value = 1.05e-111, Score = 841, Bitscore = 328.561, Identities = 158/179 (88%)</td></tr>
<tr><td>orf00009</td><td>5,876</td><td>7,009</td><td>1,134</td><td>Hypothetical protein (IbrA)</td><td>hypothetical protein [Flavobacterium psychrophilum] >gi|150024890|ref|YP_001295716.1| Protein of unknown function IbrA [Flavobacterium psychrophilum JIP02/86] >gi|149771431|emb|CAL42900.1| Protein of unknown function IbrA [Flavobacterium psychrophilum JIP02/86] >gi|697783245|gb|AIT65707.1| hypothetical protein IB65_07475 [Flavobacterium psychrophilum]</td><td>100</td><td>E-value = 0, Score = 1601, Bitscore = 621.313, Identities = 296/380 (77%)</td></tr>
<tr><td>orf00010</td><td>7,161</td><td>7,454</td><td>294</td><td>Hypothetical protein</td><td>hypothetical protein [Muricauda sp. MAR_2010_75]</td><td>2</td><td>E-value = 4.63e-12, Score = 162, Bitscore = 67.0106, Identities = 35/91 (38%)</td></tr>
<tr><td>orf00011</td><td>7,472</td><td>9,085</td><td>1,614</td><td>Phage portal protein</td><td>phage portal protein [Cellulophaga phage phiSM] >gi|460109486|gb|AGH07753.1| phage portal protein [Cellulophaga phage phiSM] >gi|514341478|gb|AGO47737.1| phage portal protein [Cellulophaga phage phi3ST:2] >gi|514341948|gb|AGO48201.1| phage portal protein [Cellulophaga phage phiSM] >gi|514343007|gb|AGO49245.1| phage portal protein [Cellulophaga phage phi38:2] >gi|514343088|gb|AGO49325.1| phage portal protein [Cellulophaga phage phi3:1] >gi|514343511|gb|AGO49743.1| phage portal protein [Cellulophaga phage phi47:1]</td><td>100</td><td>E-value = 1.53e-133, Score = 1056, Bitscore = 411.379, Identities = 210/494 (42%)</td></tr>
<tr><td>orf00012</td><td>9,099</td><td>9,332</td><td>234</td><td>NO RESULTS</td><td></td><td></td><td></td></tr>
<tr><td>orf00013</td><td>9,439</td><td>9,564</td><td>126</td><td>NO RESULTS</td><td></td><td></td><td></td></tr>
<tr><td>orf00014</td><td>9,566</td><td>9,865</td><td>300</td><td>Hypothetical protein</td><td>hypothetical protein P2559Y_0020 [Croceibacter phage P2559Y] >gi|505838236|gb|AGM14083.1| hypothetical protein P2559Y_0020 [Croceibacter phage P2559Y]</td><td>1</td><td>E-value = 6.82e-03, Score = 97, Bitscore = 41.9726, Identities = 30/84 (35%)</td></tr>
<tr><td>orf00015</td><td>9,849</td><td>10,190</td><td>342</td><td>Hypothetical protein</td><td>hypothetical protein P2559Y_0021 [Croceibacter phage P2559Y] >gi|505838237|gb|AGM14084.1| hypothetical protein P2559Y_0021 [Croceibacter phage P2559Y]</td><td>1</td><td>E-value = 1.99e-10, Score = 151, Bitscore = 62.7734, Identities = 29/62 (46%)</td></tr>
<tr><td>orf00016</td><td>10,195</td><td>10,659</td><td>465</td><td>Hypothetical protein</td><td>hypothetical protein [Sediminibacter sp. Hel_I_10]</td><td>100</td><td>E-value = 2.96e-75, Score = 594, Bitscore = 233.417, Identities = 104/151 (68%)</td></tr>
</table>

orf00017	10,662	11,006	345	Hypothetical protein	hypothetical protein PHG11b_30 [Flavobacterium phage 11b] >gi|53748196|emb|CAH56657.1| hypothetical protein PHG11b_30 [Flavobacterium phage 11b]	2	E-value = 1.07e-11, Score = 162, Bitscore = 67.0106, Identities = 41/111 (36%)
orf00018	11,171	11,413	243	NO RESULTS			
orf00019	11,415	11,798	384	Hypothetical protein	hypothetical protein Phi18:1_gp65 [Cellulophaga phage phi18:1] >gi|514342263|gb|AGO48512.1| hypothetical protein Phi18:1_gp65 [Cellulophaga phage phi18:1] >gi|514342986|gb|AGO49226.1| hypothetical protein Phi18:2_gp63 [Cellulophaga phage phi18:2]	3	E-value = 1.46e-02, Score = 98, Bitscore = 42.3578, Identities = 31/77 (40%)
**orf00020**	**12,063**	**13,325**	**1,263**	**Protease**	**hypothetical protein CEPG_00014 [Cellulophaga phage phiSM] >gi|451989184|gb|AGF91655.1| hypothetical protein CDPG_00051 [Cellulophaga phage phi47:1] >gi|460109495|gb|AGH07762.1| hypothetical protein CEPG_00014 [Cellulophaga phage phiSM] >gi|514341487|gb|AGO47746.1| Clp protease [Cellulophaga phage phi3ST:2] >gi|514341957|gb|AGO48210.1| Clp protease [Cellulophaga phage phiSM] >gi|514343016|gb|AGO49254.1| Clp protease [Cellulophaga phage phi38:2] >gi|514343097|gb|AGO49334.1| Clp protease [Cellulophaga phage phi3:1] >gi|514343520|gb|AGO49752.1| Clp protease [Cellulophaga phage phi47:1]**	**100**	**E-value = 1.05e-135, Score = 1048, Bitscore = 408.297, Identities = 217/424 (51%)**
orf00021	13,338	13,793	456	Hypothetical protein	hypothetical protein CEPG_00015 [Cellulophaga phage phiSM] >gi|451989183|gb|AGF91654.1| hypothetical protein CDPG_00050 [Cellulophaga phage phi47:1] >gi|460109496|gb|AGH07763.1| hypothetical protein CEPG_00015 [Cellulophaga phage phiSM] >gi|514341488|gb|AGO47747.1| structural protein [Cellulophaga phage phi3ST:2] >gi|514341958|gb|AGO48211.1| structural protein [Cellulophaga phage phiSM] >gi|514343017|gb|AGO49255.1| structural protein [Cellulophaga phage phi38:2] >gi|514343098|gb|AGO49335.1| structural protein [Cellulophaga phage phi3:1] >gi|514343521|gb|AGO49753.1| structural protein [Cellulophaga phage phi47:1]	2	E-value = 2.91e-23, Score = 247, Bitscore = 99.7525, Identities = 53/141 (37%)
orf00022	13,796	14,878	1,083	Hypothetical protein	hypothetical protein CEPG_00016 [Cellulophaga phage phiSM] >gi|460109497|gb|AGH07764.1| hypothetical protein CEPG_00016 [Cellulophaga phage phiSM]	58	E-value = 5.90e-143, Score = 1084, Bitscore = 422.165, Identities = 199/354 (56%)
orf00023	14,878	15,042	165	NO RESULTS			
**orf00024**	**15,057**	**15,440**	**384**	**Structural protein**	**hypothetical protein CEPG_00018 [Cellulophaga phage phiSM] >gi|451988698|gb|AGF91174.1| hypothetical protein CHPG_00022 [Cellulophaga phage phi3:1] >gi|451989180|gb|AGF91651.1| hypothetical protein CDPG_00047 [Cellulophaga phage phi47:1] >gi|460109499|gb|AGH07766.1| hypothetical protein CEPG_00018 [Cellulophaga phage phiSM] >gi|514341491|gb|AGO47750.1| structural protein [Cellulophaga phage phi3ST:2] >gi|514341961|gb|AGO48214.1| structural protein [Cellulophaga phage phiSM] >gi|514343020|gb|AGO49258.1| structural protein [Cellulophaga phage phi38:2] >gi|514343101|gb|AGO49338.1| structural protein [Cellulophaga phage phi3:1] >gi|514343524|gb|AGO49756.1| structural protein [Cellulophaga phage phi47:1]**	**2**	**E-value = 1.57e-34, Score = 319, Bitscore = 127.487, Identities = 58/126 (46%),**
orf00025	15,437	16,042	606	Hypothetical protein	hypothetical protein [Muricauda sp. MAR_2010_75]	5	E-value = 3.72e-39, Score = 363, Bitscore = 144.436, Identities = 78/199 (39%)
**orf00026**	**16,048**	**17,568**	**1,521**	**Tail sheath protein**	**tail protein [Cellulophaga phage phiSM] >gi|451989178|gb|AGF91649.1| tail protein [Cellulophaga phage phi47:1] >gi|460109501|gb|AGH07768.1| tail protein [Cellulophaga phage phiSM] >gi|514341493|gb|AGO47752.1| tail sheat protein [Cellulophaga phage phi3ST:2] >gi|514341963|gb|AGO48216.1| tail sheat protein [Cellulophaga phage phiSM] >gi|514343022|gb|AGO49260.1| tail sheat protein [Cellulophaga phage phi38:2] >gi|514343103|gb|AGO49340.1| tail sheat protein [Cellulophaga phage phi3:1] >gi|514343526|gb|AGO49758.1| tail sheat protein [Cellulophaga phage phi47:1]**	**100**	**E-value = 0, Score = 1638, Bitscore = 635.565, Identities = 312/508 (61%)**
**orf00027**	**17,589**	**17,999**	**411**	**Structural protein**	**hypothetical protein CEPG_00021 [Cellulophaga phage phiSM] >gi|451988704|gb|AGF91180.1| hypothetical protein CHPG_00028 [Cellulophaga phage phi3:1] >gi|451989177|gb|AGF91648.1| hypothetical protein CDPG_00044 [Cellulophaga phage phi47:1] >gi|460109502|gb|AGH07769.1| hypothetical protein CEPG_00021 [Cellulophaga phage phiSM] >gi|514341494|gb|AGO47753.1| structural protein [Cellulophaga phage phi3ST:2] >gi|514343023|gb|AGO49261.1| structural protein [Cellulophaga phage phi38:2] >gi|514343104|gb|AGO49341.1| structural protein [Cellulophaga phage phi3:1] >gi|514343527|gb|AGO49759.1| structural protein [Cellulophaga phage phi47:1]**	**4**	**E-value = 1.36e-44, Score = 388, Bitscore = 154.066, Identities = 70/136 (51%)**
orf00028	18,055	18,471	417	Hypothetical protein	hypothetical protein [Muricauda sp. MAR_2010_75]	1	E-value = 8.94e-06, Score = 121, Bitscore = 51.2174, Identities = 41/140 (29%)
orf00029	18,719	20,548	1,83	Hypothetical protein	hypothetical protein [Riemerella anatipestifer] >gi|407452085|ref|YP_006723810.1| hypothetical protein B739_1312 [Riemerella anatipestifer RA-CH-1] >gi|403313069|gb|AFR35910.1| hypothetical protein B739_1312 [Riemerella anatipestifer RA-CH-1]	100	E-value = 1.11e-44, Score = 462, Bitscore = 182.57, Identities = 118/348 (33%)

orf00030	20,564	21,043	480	Hypothetical protein	hypothetical protein CEPG_00025 [Cellulophaga phage phiSM] >gi|451989173|gb|AGF91644.1| hypothetical protein CDPG_00040 [Cellulophaga phage phi47:1] >gi|460109506|gb|AGH07773.1| hypothetical protein CEPG_00025 [Cellulophaga phage phiSM] >gi|514341498|gb|AGO47757.1| structural protein [Cellulophaga phage phi3ST:2] >gi|514341968|gb|AGO48221.1| structural protein [Cellulophaga phage phiSM] >gi|514343027|gb|AGO49265.1| structural protein [Cellulophaga phage phi38:2] >gi|514343108|gb|AGO49345.1| structural protein [Cellulophaga phage phi3:1] >gi|514343531|gb|AGO49763.1| structural protein [Cellulophaga phage phi47:1]	3	E-value = 3.86e-48, Score = 416, Bitscore = 164.851, Identities = 82/158 (51%)
orf00031	21,049	22,224	1,176	Baseplate J-like protein	hypothetical protein CEPG_00026 [Cellulophaga phage phiSM] >gi|451989172|gb|AGF91643.1| hypothetical protein CDPG_00039 [Cellulophaga phage phi47:1] >gi|460109507|gb|AGH07774.1| hypothetical protein CEPG_00026 [Cellulophaga phage phiSM] >gi|514341499|gb|AGO47758.1| baseplate J-like protein [Cellulophaga phage phi3ST:2] >gi|514341969|gb|AGO48222.1| baseplate J-like protein [Cellulophaga phage phiSM] >gi|514343028|gb|AGO49266.1| baseplate J-like protein [Cellulophaga phage phi38:2] >gi|514343109|gb|AGO49346.1| baseplate J-like protein [Cellulophaga phage phi3:1] >gi|514343532|gb|AGO49764.1| baseplate J-like protein [Cellulophaga phage phi47:1]	100	E-value = 1.61e-140, Score = 1074, Bitscore = 418.313, Identities = 215/390 (55%)
orf00032	22,229	23,008	780	Tail protein	hypothetical protein CEPG_00027 [Cellulophaga phage phiSM] >gi|451989171|gb|AGF91642.1| hypothetical protein CDPG_00038 [Cellulophaga phage phi47:1] >gi|460109508|gb|AGH07775.1| hypothetical protein CEPG_00027 [Cellulophaga phage phiSM] >gi|514341500|gb|AGO47759.1| putative tail protein [Cellulophaga phage phi3ST:2] >gi|514341970|gb|AGO48223.1| putative tail protein [Cellulophaga phage phiSM] >gi|514343029|gb|AGO49267.1| putative tail protein [Cellulophaga phage phi38:2] >gi|514343110|gb|AGO49347.1| putative tail protein [Cellulophaga phage phi3:1] >gi|514343533|gb|AGO49765.1| putative tail protein [Cellulophaga phage phi47:1]	29	E-value = 4.73e-79, Score = 640, Bitscore = 251.136, Identities = 121/253 (47%)
orf00033	23,031	24,077	1,047	Structural protein	hypothetical protein CEPG_00028 [Cellulophaga phage phiSM] >gi|451988684|gb|AGF91160.1| hypothetical protein CHPG_00008 [Cellulophaga phage phi3:1] >gi|451989170|gb|AGF91641.1| hypothetical protein CDPG_00037 [Cellulophaga phage phi47:1] >gi|460109509|gb|AGH07776.1| hypothetical protein CEPG_00028 [Cellulophaga phage phiSM] >gi|514341501|gb|AGO47760.1| structural protein [Cellulophaga phage phi3ST:2] >gi|514341971|gb|AGO48224.1| structural protein [Cellulophaga phage phiSM] >gi|514343030|gb|AGO49268.1| structural protein [Cellulophaga phage phi38:2] >gi|514343111|gb|AGO49348.1| structural protein [Cellulophaga phage phi3:1] >gi|514343534|gb|AGO49766.1| structural protein [Cellulophaga phage phi47:1]	6	E-value = 5.66e-58, Score = 515, Bitscore = 202.986, Identities = 108/241 (44%)
orf00034	24,081	25,250	1,17	Hypothetical protein	hypothetical protein [Muricauda sp. MAR_2010_75]	5	E-value = 2.84e-66, Score = 578, Bitscore = 227.254, Identities = 138/395 (34%)
orf00035	25,247	25,906	660	Structural protein	hypothetical protein CDPG_00035 [Cellulophaga phage phi47:1] >gi|514341503|gb|AGO47762.1| structural protein [Cellulophaga phage phi3ST:2] >gi|514341973|gb|AGO48226.1| structural protein [Cellulophaga phage phiSM] >gi|514343032|gb|AGO49270.1| structural protein [Cellulophaga phage phi38:2] >gi|514343113|gb|AGO49350.1| structural protein [Cellulophaga phage phi3:1] >gi|514343536|gb|AGO49768.1| structural protein [Cellulophaga phage phi47:1]	5	E-value = 2.76e-36, Score = 349, Bitscore = 139.043, Identities = 94/249 (37%)
orf00036	25,903	27,117	1,215	Structural protein	hypothetical protein CEPG_00032 [Cellulophaga phage phiSM] >gi|451989167|gb|AGF91638.1| hypothetical protein CDPG_00034 [Cellulophaga phage phi47:1] >gi|460109513|gb|AGH07780.1| hypothetical protein CEPG_00032 [Cellulophaga phage phiSM] >gi|514341504|gb|AGO47763.1| structural protein [Cellulophaga phage phi3ST:2] >gi|514341974|gb|AGO48227.1| structural protein [Cellulophaga phage phiSM] >gi|514343033|gb|AGO49271.1| structural protein [Cellulophaga phage phi38:2] >gi|514343114|gb|AGO49351.1| structural protein [Cellulophaga phage phi3:1] >gi|514343537|gb|AGO49769.1| structural protein [Cellulophaga phage phi47:1]	7	E-value = 6.66e-87, Score = 720, Bitscore = 281.952, Identities = 160/409 (39%)
orf00037	27,120	28,166	1,047	Tail protein	hypothetical protein CEPG_00033 [Cellulophaga phage phiSM] >gi|451989166|gb|AGF91637.1| tail protein [Cellulophaga phage phi47:1] >gi|460109514|gb|AGH07781.1| hypothetical protein CEPG_00033 [Cellulophaga phage phiSM] >gi|514341505|gb|AGO47764.1| putative tail protein [Cellulophaga phage phi3ST:2] >gi|514341975|gb|AGO48228.1| putative tail protein [Cellulophaga phage phiSM] >gi|514343034|gb|AGO49272.1| putative tail protein [Cellulophaga phage phi38:2] >gi|514343115|gb|AGO49352.1| putative tail protein [Cellulophaga phage phi3:1] >gi|514343538|gb|AGO49770.1| putative tail protein [Cellulophaga phage phi47:1]	100	E-value = 6.05e-83, Score = 682, Bitscore = 267.314, Identities = 142/336 (42%)
orf00038	28,169	28,657	489	Hypothetical protein	hypothetical protein CHPG_00033 [Cellulophaga phage phi3:1] >gi|451989165|gb|AGF91636.1| hypothetical protein CDPG_00032 [Cellulophaga phage phi47:1] >gi|514341506|gb|AGO47765.1| structural protein [Cellulophaga phage phi3ST:2] >gi|514341976|gb|AGO48229.1| structural protein [Cellulophaga phage phiSM] >gi|514343035|gb|AGO49273.1| structural protein [Cellulophaga phage phi38:2] >gi|514343116|gb|AGO49353.1| structural protein [Cellulophaga phage phi3:1] >gi|514343539|gb|AGO49771.1| structural protein [Cellulophaga phage phi47:1]	10	E-value = 9.54e-20, Score = 225, Bitscore = 91.2781, Identities = 47/124 (37%)

orf00039	28,817	29,059	243	Hypothetical protein	PREDICTED: kinesin-like protein KIF21B isoform X6 [Esox lucius]	18	E-value = 3.91e-02, Score = 97, Bitscore = 41.9726, Identities = 20/47 (42%)
orf00040	29,203	29,499	297	Hypothetical protein	hypothetical protein Phi19:3_gp062 [Cellulophaga phage phi19:3] >gi|514341204|gb|AGO47466.1| hypothetical protein Phi19:3_gp062 [Cellulophaga phage phi19:3]	3	E-value = 2.29e-08, Score = 139, Bitscore = 58.151, Identities = 33/94 (35%)
orf00041	29,663	29,959	297	Hypothetical protein	hypothetical protein [Hassallia byssoidea] >gi|746975558|gb|KIF32209.1| hypothetical protein PI95_39980 [Hassallia byssoidea VB512170]	1	E-value = 1.31e-02, Score = 100, Bitscore = 43.1282, Identities = 28/97 (28%)
orf00042	30,066	30,302	237	NO RESULTS			
orf00043	30,305	30,565	261	NO RESULTS			
orf00044	30,568	30,753	186	NO RESULTS			
orf00045	30,773	31,933	1,161	Hypothetical protein	hypothetical protein [Leeuwenhoekiella sp. Hel_I_48]	3	E-value = 2.19e-33, Score = 350, Bitscore = 139.428, Identities = 110/320 (34%)
orf00046	31,936	32,304	369	NO RESULTS			
orf00047	32,318	33,073	756	Hypothetical protein	hypothetical protein, partial [Clostridium sp. K25]	100	E-value = 6.52e-63, Score = 530, Bitscore = 208.764, Identities = 108/218 (49%)
orf00048	33,060	33,413	354	Hypothetical protein	nucleoside triphosphate pyrophosphohydrolase, MazG [Cellulophaga phage phi19:3] >gi|514341214|gb|AGO47476.1| nucleoside triphosphate pyrophosphohydrolase, MazG [Cellulophaga phage phi19:3]	100	E-value = 1.43e-22, Score = 237, Bitscore = 95.9005, Identities = 50/115 (43%)
orf00049	33,478	33,615	138	NO RESULTS			
orf00050	33,615	34,358	744	Hypothetical protein	hypothetical protein, partial [Chryseobacterium daeguense]	1	E-value = 1.71e-03, Score = 113, Bitscore = 48.1358, Identities = 48/173 (27%)
**orf00051**	**34,351**	**36,876**	**2,526**	**DNA methylase**	**DNA methylase N-4 [Treponema sp. OMZ 838]**	**100**	**E-value = 0, Score = 2092, Bitscore = 810.446, Identities = 397/835 (47%)**
orf00052	36,857	37,225	369	NO RESULTS			
orf00053	37,218	37,469	252	NO RESULTS			
orf00054	37,596	38,312	717	Hypothetical protein	hypothetical protein [Pseudomonas putida] >gi|428763530|gb|EKX85699.1| hypothetical protein CSV86_08191 [Pseudomonas putida CSV86]	100	E-value = 1.55e-23, Score = 265, Bitscore = 106.686, Identities = 55/113 (48%)
orf00055	38,411	39,364	954	Hypothetical protein	TOPRIM domain-containing protein [Phaeospirillum fulvum MGU-K5]	100	E-value = 1.04e-22, Score = 270, Bitscore = 108.612, Identities = 77/277 (27%)
orf00056	39,376	39,819	444	NO RESULTS			
orf00057	39,824	40,009	186	NO RESULTS			
orf00058	40,013	40,534	522	NO RESULTS			
orf00059	40,629	41,153	525	Hypothetical protein	hypothetical protein [Flavobacterium phage FCV-1]	1	E-value = 1.29e-119, Score = 891, Bitscore = 347.821, Identities = 171/174 (98%)
orf00060	41,210	41,437	228	Hypothetical protein	hypothetical protein [Chitinophaga pinensis] >gi|256422896|ref|YP_003123549.1| hypothetical protein Cpin_3886 [Chitinophaga pinensis DSM 2588] >gi|256037804|gb|ACU61348.1| hypothetical protein Cpin_3886 [Chitinophaga pinensis DSM 2588]	17	E-value = 4.55e-16, Score = 186, Bitscore = 76.2554, Identities = 37/69 (53%)
orf00061	41,453	41,887	435	Hypothetical protein	MULTISPECIES: hypothetical protein [Myroides] >gi|373909651|gb|EHQ41500.1| hypothetical protein Myrod_0664 [Myroides odoratus DSM 2801] >gi|404603027|gb|EKB02707.1| hypothetical protein HMPREF9716_03736 [Myroides [odoratimimus] CIP 103059]	3	E-value = 2.27e-25, Score = 258, Bitscore = 103.99, Identities = 53/93 (56%)
orf00062	41,884	42,027	144	NO RESULTS			
orf00063	42,032	42,478	447	Hypothetical protein	single-stranded DNA-binding protein [Sediminibacterium sp. C3]	100	E-value = 4.61e-14, Score = 183, Bitscore = 75.0998, Identities = 43/148 (29%)
orf00064	42,600	42,998	399	NO RESULTS			

orf00065	43,049	43,348	300	Hypothetical protein	hypothetical protein [Flavobacterium columnare] >gi|365960114|ref|YP_004941681.1| hypothetical protein FCOL_05315 [Flavobacterium columnare ATCC 49512] >gi|365736795|gb|AEW85888.1| hypothetical protein FCOL_05315 [Flavobacterium columnare ATCC 49512]	1	E-value = 7.74e-44, Score = 386, Bitscore = 153.295, Identities = 79/99 (79%)
orf00066	43,599	43,736	138	NO RESULTS			
orf00067	43,767	44,015	249	NO RESULTS			
orf00068	44,050	44,262	213	Hypothetical protein	hypothetical protein P2559S_07 [Croceibacter phage P2559S] >gi|392284355|gb|AFM54785.1| hypothetical protein P2559S_07 [Croceibacter phage P2559S]	1	E-value = 7.82e-03, Score = 96, Bitscore = 41.5874, Identities = 19/46 (41%)
orf00069	44,277	44,546	270	NO RESULTS			
orf00070	44,709	45,056	348	NO RESULTS			
orf00071	45,363	45,806	444	Hypothetical protein	hypothetical protein [Flavobacterium indicum] >gi|383451233|ref|YP_005357954.1| hypothetical protein KQS_09835 [Flavobacterium indicum GPTSA100-9 = DSM 17447] >gi|380502855|emb|CCG53897.1| Protein of unknown function precursor [Flavobacterium indicum GPTSA100-9 = DSM 17447]	51	E-value = 1.25e-05, Score = 124, Bitscore = 52.373, Identities = 28/57 (49%)
orf00072	45,809	46,195	387	Hypothetical protein	phage_TIGR01671: phage conserved hypothetical protein TIGR01671 [Desulfitobacterium hafniense]	100	E-value = 6.39e-15, Score = 188, Bitscore = 77.0258, Identities = 50/146 (34%)
orf00073	46,330	46,671	342	NO RESULTS			
orf00074	46,674	46,985	312	Hypothetical protein	hypothetical protein Phi19:1_gp075 [Cellulophaga phage phi19:1] >gi|514341102|gb|AGO47365.1| hypothetical protein Phi19:1_gp075 [Cellulophaga phage phi19:1]	2	E-value = 1.22e-30, Score = 288, Bitscore = 115.546, Identities = 52/76 (68%)
